# Proteomic and genomic biomarkers for Non‐Small Cell Lung Cancer: Peroxiredoxin, Haptoglobin, and Alpha‐1 antitrypsin

**DOI:** 10.1002/cam4.3019

**Published:** 2020-03-30

**Authors:** Zahra Najafi, Abdolreza Mohamadnia, Rahim Ahmadi, Minoo Mahmoudi, Naghmeh Bahrami, Adnan Khosravi, Hamidreza Jamaati, Payam Tabarsi, Mehdi Kazem pour Dizaji, Sadegh Shirian

**Affiliations:** ^1^ Department of Biology Faculty of Basic Sciences Hamedan Branch Islamic Azad University Hamedan Iran; ^2^ Chronic Respiratory Diseases Research Center National Research Institute of Tuberculosis and Lung Diseases (NRITLD) Shahid Beheshti University of Medical Sciences Tehran Iran; ^3^ Department of Biotechnology School of Advanced Technologies in Medicine Shahid Beheshti University of Medical Sciences Tehran Iran; ^4^ Department of Tissue Engineering and Applied Cell Sciences School of Advanced Technologies in Medicine Tehran University of Medical Sciences Tehran Iran; ^5^ Craniomaxillofacial Research Center School of Dentistry Tehran University of Medical Sciences Tehran Iran; ^6^ Tobacco Prevention and Control Research Center National Research Institute of Tuberculosis and Lung Diseases (NRITLD) Shahid Beheshti University of Medical Sciences Tehran Iran; ^7^ Clinical Tuberculosis and Epidemiology Research Center National Research Institute of Tuberculosis and Lung Diseases (NRITLD) Shahid Beheshti University of Medical Sciences Tehran Iran; ^8^ Biostatistics Department Mycobacteriology Research Center National Research Institute of Tuberculosis and Lung Diseases Masih Daneshvari Hospital Shahid Beheshti University of Medical Sciences Tehran Iran; ^9^ Department of Pathology School of Veterinary Medicine Shahrekord University Shahrekord Iran; ^10^ Shiraz Molecular Pathology Research Center Dr Daneshbod Lab Shiraz Iran; ^11^ Shefa Neuroscience Research Center Tehran Iran

**Keywords:** Alpha‐1 antitrypsin, Antioxidant, Haptoglobin, lung cancer, NSCLC, Peroxiredoxin-2, proteomics, tumor markers

## Abstract

**Background:**

The development of lung cancer is a multifactorial process that involves the environmental and genetic factors. The mortality rate of this cancer is higher than breast, colorectal, and prostate cancers. In this study, we try to analyze the proteome of patients with Non‐Small Cell Lung Cancer (NSCLC) and compare it with the healthy samples.

**Methods:**

This study has compared 30 lung tissue samples from patients with NSCLC and 30 healthy samples using proteomics and RT‐PCR. Hence, tissue samples were obtained from the surgical ward in sterile conditions, and then, protein extraction applied to them. At the next stage, two‐dimensional electrophoresis and mass spectrometry LCMS/MS were performed for protein isolation and sequencing, respectively.

**Results:**

The proteome analysis identified more than 40 differences in proteomic pattern of normal lung tissues compared to lung tissues with NSCLC. Peroxiredoxin, Haptoglobin, and Alpha‐1 antitrypsin proteins were identified. Molecularly, it has also been shown that the two main proteins of Peroxiredoxin‐2 and Alpha‐1 antitrypsin were upregulated, and the expression of Haptoglobin protein was downregulated in cancer tissue.

**Conclusion:**

The results of this study showed that there are some differences in term of protein content between the normal and cancerous lung tissues. Further studies are needed to evaluate these proteins that investigate whether these proteins can candidate as biomarkers to use in the early diagnosis of patients with NSCLC.

## Graphical abstract

Considering the results of past research and our current study, we find that the expression of two major proteins of Peroxiredoxin‐2 and Alpha‐1 antitrypsin is dramatically decreased in the cancerous tissues, and the expression level of Haptoglobin in the cancerous tissue is strongly increased.

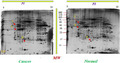

## 1 INTRODUCTION

Lung cancer (LC) is the most common cancer worldwide and lead to most cancer‐related mortality in the world (Hoseok). It is the leading cause of death among cancers in men and is the second leading cause of death in women after the breast cancer.^1^, ^2^ Genes that cause the growth and proliferation phenotypes are called oncogenes, and those that regulate or inhibit proliferation are tumor‐suppressor genes. These genes actually induce the degree of cell proliferation, differentiation‐inducing, regulating cellular communication with adjacent cells, and surrounding tissue cells, induced premature aging or death.^3^, ^4^ Tumor suppressor genes encode proteins that play a role to stop the tumor growth and formation. When mutations cause loss of function of these genes, there is a lack of control and growth inhibition, thereby allowing growth.^5^ Symptoms of LC are not usually recognized until the cancer is in an advanced and non‐curable state. Late diagnosis is a main factor contributing to the poor LC prognosis. Therefore, the early detection of biomarkers for effective prognosis is of utmost importance.^6^ Non‐small cell lung cancer is a lethal disease that is still among the most common causes of cancer deaths in the world. LC is a type of lung disease that its feature is uncontrolled cell growth in lung tissue. If disease left untreated, cell growth can metastasize outside the lungs and neighboring tissues or other organs. Basal epithelial cell changes and the lung epithelium are the origins of more than 90% of LCs. LC occurs when the lung cells grow uncontrollably, they are able to become tumors and spread to other parts of the body.^7^ Proteomic analysis has recently became an integral approach for investigation of functional tumor biology of malignant tumors such as NSCLS, complementing the genetic analysis. Proteomics also evaluates the expressions, functions, and interactions of proteins.^8^ The peroxiredoxin protein family is involved in the cancer cell invasion and metastasis and prognostic role of peroxiredoxin proteins in LC has been recently demonstrated.^9^ However their prognostic values in NSCLC remain unknown. The increasing of haptoglobin in serum of patients with NSCLC was potentially useful in the clinical diagnosis of this cancer, especially in male subjects.^10^ Alpha‐1 antitrypsin is a glycoprotein that inhibits the serine protease and acts as a restrictor of cellular injury and death in tissue. Although, its antiapoptotic role has been confirmed.^11^ Upregulation of *SERPINA1* gene encoding acute phase protein, alpha‐1 antitrypsin, is associated with various tumors. *SERPINA1* gene and Alpha‐1 antitrypsin protein have been recently shown to play an active role in the pathogenesis of LC such as NSCLS and not just reflect inflammatory reaction related to the cancer development.^12^ Therefore, this study aimed to investigate whether the quantitative amount of Peroxiredoxin‐2 proteins that are candidates of tumor suppressor role as well as Haptoglobin and Alpha‐1 antitrypsin in patients with NSCLS can be biomarker candidate to early diagnosis of this cancer.

## 2 MATERIALS AND METHODS

### 2.1 Collecting and maintaining patients’ normal and cancerous tissues

All patients provided their written, informed consent, and the samples were then obtained. The normal and cancerous tissue of each patient was immediately collected into sterile gas after surgery, and transferred to a container containing the liquid air, and maintained until the extraction of proteins. The data of each patient such as pathology report and the degree of metastasis were recorded.

### 2.2 Total protein extraction from lung tissues

The total proteins of lung tissues were extracted according to previous studies.^13^, ^14^, ^15^ Normal and tumor tissues were cut to 150‐200 mg within a petri dish containing liquid nitrogen, and the tissues were completely powdered by placing in a mortar containing liquid air, and this was repeated several times. Then, Tris buffer at pH = 7, 20 M Tris‐Hcl, 8 mol/L urea, 2 mol/L thiourea, 2% CHAPS, 50 mmol/L DTT, 4% PMSF, and 2% DNAase I were added to the samples, vortexed for 30 minutes; then centrifuged at 4°C at 13 000 rpm for 30 minutes, and the supernatant was removed, and maintained for protein assay experiments, and the residue was kept −70°C. About 5 µL of supernatant was used to measure the protein concentration by Bradford method.^16^

### 2.3 Two‐dimensional electrophoresis

The first dimension of the ready‐made strip, purchased from Biorad Co., was used at a suitable pH of 3‐10, and the extracted samples were placed on it, and isoelectric focusing was done at 600 V, for 1 hour, and then, 1000 V for 1 hour. At the end of the time, the gels were slowly put into modulator buffer containing 600 mmol/L Tris‐Chloric acid (or base), 2% sodium dodecyl sulfate, 5% beta‐Mercaptoethanol, 10% glycerol at pH 6.8, and 0.002% bromophenol blue, and after 20 minutes, the first dimension gel was removed from the solution, and then, applied to the second‐dimension gel, which is SDS‐PAGE.

### 2.4 Protein staining through silver nitrate staining, in which two modified Bloom methods were used to staining

Proteins were detected as previously described by Nesterenko et al (1994) with a slight modification. In brief, the gel was fixed for at least 1 h with constant shaking and followed by washing with 50% ethanol, pretreated with sodium thiosulfate for 1 minute, and washed three times with ddH2O. The gel was Impregnated with AgNO3 and the residual AgNO3 was removed by 3 × 20 s successive washed with deionized water. The gel was developed by soaking it in developing solution containing Na_2_CO_3_, formaldehyde and Na_2_S_2_O_3_ for 10 minutes up until it appeared to develop yellowish brown spots. The gel was then rinsed twice, each for 2 minutes, with ddH2O. Further development was stopped by immersing the gel in stop solution (50% methanol and 12% acetic acid) and stored in 30% ethanol at 4°C until scanning.^17^, ^18^

### 2.5 Identifying the characteristics of each polypeptide spot

To specify the polypeptide status on the gel plate, the normal and tumor tissue samples from the same case was compared. The location of each spot, its size, and its severity were used to the qualitative and quantitative comparison of gels of normal and tumor tissues. Qualitative and quantitative comparisons between two gels were made based on the proposed method and by eye‐tracking. These variations are removing a spot, the appearance of a new spot, increasing, or decreasing the intensity of the spot. The differences between each pair of gels (normal and tumor) are noted in the special table. With proteome analysis of all patients' samples, those polypeptides spots that showed 70% or more changes such as deletion, amplification or qualified changes were identified and reported as final results.

### 2.6 Mass spectrometry

After detecting the desired differences of two‐dimensional gels, proteins were carefully removed from the gel and put in the 0.5 mL vial containing 5% acetic acid, and by SINACLON Co. and through LCMS/MS in order to polypeptide detection were sent to Canada. Acquisition was performed with an ABSciexTripleTOF 5600 (ABSciex) equipped with an electrospray interface with a 25 μm iD capillary and coupled to an EksigentμUHPLC (Eksigent). Analyst TF 1.7 software was used to control instrument as well as data processing and acquisition.

### 2.7 Bioinformatics

The results of mass spectrometry were analyzed and evaluated by databases such as Expassy, Profound, ebi, pdb, malsoft, and etc.

### 2.8 Real‐time RT‐PCR

A total of 10 mg of fresh tissue were cut, homogenized, and transferred into a 1.5 mL RNase‐DNase free micro‐tube. The steps of RNA extraction were performed using RNA extraction kit according to the instructions in the kit (Cinna pure Cat no. PR891620). The RNA of the samples were evaluated by a NanoDrop, as well as their absorbance were measured by a spectrophotometer, in a wavelength of 260‐280 nm, and their ratio in the range of 1.8‐2 were acceptable. cDNA was synthesized using Viva 2‐steps RT‐PCR Kit (Cat no. RTPL12) according to the available protocol in the kit. The main parts to Reverse Transcription were included in the RT Primer Mix and Reverse Transcriptase has been provided in the kit. Dedicated primers for each marker were prepared and ordered to manufacture. Reference gene primers (18s rRNA) were also prepared simultaneously. The characteristics of the primers used in the Real‐time RT‐PCR reaction are presented in Table 1.

**TABLE 1:** The characteristics of the primers used in the real‐time RT‐PCR reaction

Parameters	18s rRNA	Alpha‐1 antitrypsin	Haptoglobin	Peroxiredoxin‐2
Initiator F	GTAACCCGTTGAACCCCATT	ATAAGGCTGTGCTGACCATCGTC	TCAGTGTCACCATGATTATCCA	CCAGACGCTTGTCTGAGGAT
Length of primer	20	23	23	21
Initiator R	CCATCCAATCGGTAGTAGCG	TTGGGTGGGATTCACCACTTTTC	GATTTAACACACTAAGCCCTTTGG	ACGTTGGGCTTAATCGTGTC
Length of primer	20	23	20	20

Real‐time RT‐PCR reaction was performed by the Kit of Sinacolon Co. (Cat No. MM2041). The necessary elements to do the reaction in the master‐mix kit have been provided. The elements of the Real‐time RT‐PCR reaction were:

A. cDNA, 2 µL, B. Master Mix, 4 µL, C. Primer, considering the most appropriate found concentration in the initial set‐up experiments, D. Deionized distilled water, to the extent that the last reaction volume reaches 20 µL.

The reaction temperatures and times were set up according to the kit instructions (Table 2). At the end of each reaction, the results’ interpretation provided based on the amplification and melting peak curves.

**TABLE 2:** Temperatures and times of real‐time RT‐PCR reaction

Real‐time step	Temperature	Duration
Initial activation	95°C	10 min
40 cycles of		
Denaturation	95°C	15 s
Annealing	56‐60°C	60 s
Extension	72°C	20 s

In order to examine the relative difference of gene expression in the two research groups, ∆∆Ct that is the common method of analyzing the gene expression differences in Real‐time RT‐PCR, was used. Marker C*_t_* values and reference gene C*_t_* values (which is used to normalize) in each sample were extracted from the mentioned test results and finally, research ∆∆C*_t_* was obtained. Now if we raise the number 2 to the power of ∆∆C*_t_* the difference of marker expression will be determined.

## 3 RESULTS

### 3.1 Specifying the pathologic status of patients

In this study, to get effective molecular markers in lung cancer, tissue samples of 30 patients, 2 cm normal and 2 cm tumor tissues, were examined, and accessibility to pathological records of all these patients became possible by the Department of Pathology. This group of the patient included 11 female and 19 male patients, and the age range of them was 23‐73 years, and mean age of 58 years.

### 3.2 Proteomics study

The main aspect of this study is that there are many genetic products in various cancers that their expression may be upregulated or downregulated or deleted compared to the normal cells. Therefore, it is necessary to use a method that be able to study many of the genetic products, polypeptides, at the same time. To this end, the best method is two‐dimensional electrophoresis, which is an essential part of such research. In the first dimension, the separation of proteins is done through IEF and in the second dimension based on the molecular weight (MW) via electrophoresesis in acrylamide gel (SDS‐PAGE). The status of electrophoresis in these two dimensions is perpendicular. Each spot may contain one or more polypeptides with pI and very close MW, which in this case it is better to use more accurate pI range in the first dimension.

**FIGURE 1:**
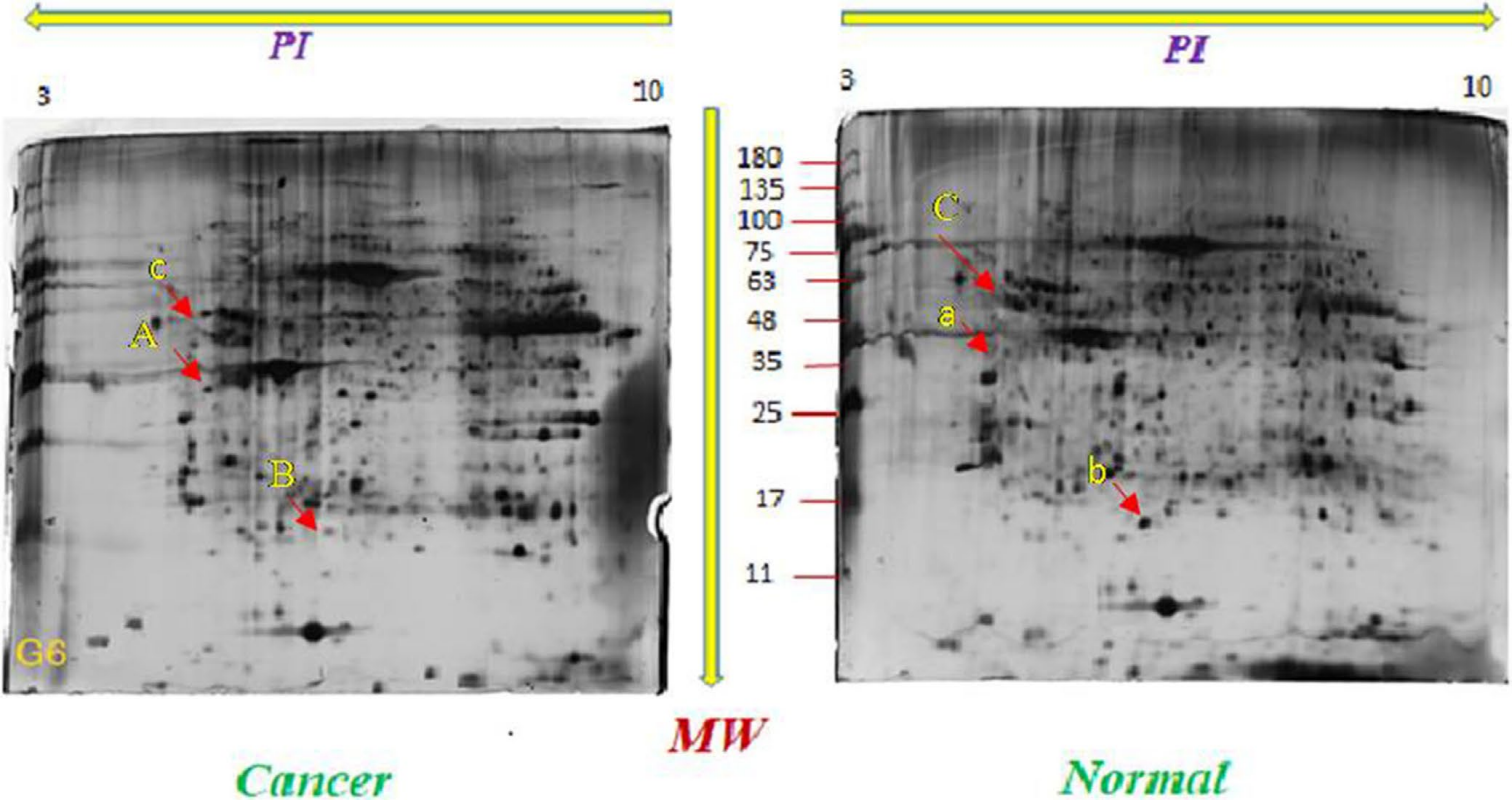
The pattern of polypeptide spot in 70% (or more) of the second‐dimension gel of the patients’ cancerous tissue showed increasing or decreasing expression compared to normal tissue is observed. Arrows and numbers indicate downregulated proteins compared to their matched tumor tissue. Qualified changes is determined by A, B, and C. A molecule has upregulated, whereas B and C molecules have downregulated

It should be noted that all experiments, the loaded amount of total protein in each tissue, in the first dimension, have been taken in to account 75 µgr. The pattern of polypeptide spot in 70% (or more) of the second‐dimension gel of the patients’ cancerous tissue showed increasing or decreasing expression compared to normal tissue is observed in Figure 1.

The increasing of protein Ain tumor tissues compared to their corresponding normal tissues suggests that upregulation of a protein contributes to the development of malignancy. The decreased proteins in tumoral tissues compared to their corresponding normal tissues, are labeled alphabetically as B, C were considerably downregulate. It suggests that downregulation if the proteins are involved in the maintenance of normal phenotype.

In this study, several differences between normal and cancerous tissue gels were used for mass spectrometry; this paper discusses three of them.

### 3.3 Studying the samples by mass spectrometry

After determining and specifying the location of desired polypeptides on the two‐dimensional gel plates, the spots were carefully removed and were put in 0.5 mL vials, then their mass spectrometry sent as LCMS/MS. It is worth noting that, from each spot of the specimens in Figure 1, three corresponding specimens were sent to make sure the correct detection of the spots; fortunately, digestion of duplicate specimens showed the same reaction.

Considering the analysis of above‐mentioned spots in the databases, three main proteins including Haptogbolin, Peroxiredoxin‐2, and Alpha‐1 antitrypsin were selected, and some attributes of these spots such as their Accession numbers in NCBI database, Location, and expression profiling are presented in Table 3.

**TABLE 3:** Mass spectrometric identification and characteristics of the three proteins whose expression were subjected to change in Non‐Small Cell Lung Cancer

Polypeptide	Protein name	Tissue specification	Accession number based on NCBI	Chromosome	Expression profiling or level
A	Haptogbolin	Tumor	sp|Q13162|PRDX2	19p13.13	Up regulation
B	Peroxiredoxin‐2	Normal	sp|P00738|HPT	16q22.2	Downregulation
C	Alpha‐1 antitrypsin	Normal	sp|P13998|AAT_H UMAN	14q32.13	Downregulation

### 3.4 Results of real‐time RT‐PCR studies

Three constructed cDNA vials were examined for each patient to express the reference genes and markers. The interpretation of results was done by the ∆∆C*_t_* method and based on the Melting peak curve. After extracting the results of real‐time RT‐PCR reaction, the mean positive percentage of each markers in normal and cancerous samples were determined. Out of 30 samples, peroxiredoxin‐2 mRNA marker was positive in 5 and 25 cancerous and normal sample, respectively. Statistical comparison of the positive marker between two groups has been done using Two‐sample binomial test and indicates a statistically significant difference between these two groups (*P* < .001).

Form 30 samples, Alpha‐1 antitrypsin mRNA marker was positive in nine cancerous and normal samples, respectively. Statistical comparison of the positive marker between two groups has been done using Two‐sample binomial test, which indicates a statistically significant difference between these two groups (*P* < .001).

**FIGURE 2:**
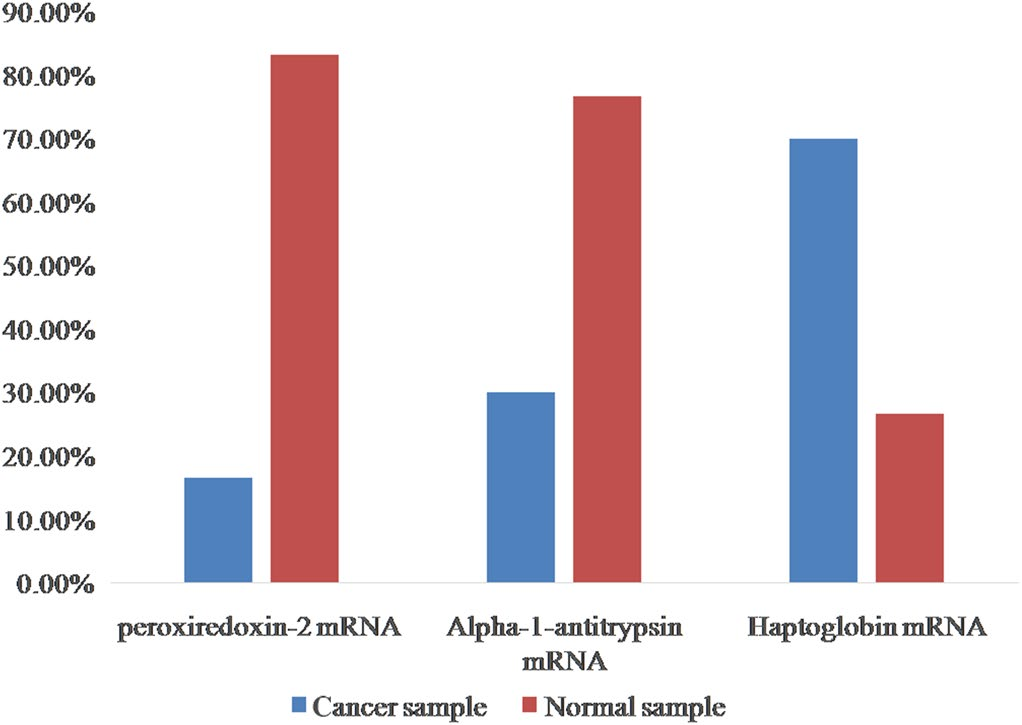
The positive percentage of Peroxiredoxin‐2, Alpha‐1 antitrypsin, and Haptoglobin markers in normal and cancerous tissue

Out of 30 samples, Haptoglobin marker was positive in 21 cancer and normal samples, respectively. Statistical comparison of the positive marker between the two groups has been done using two‐sample binomial test, which indicates a statistically significant difference between these two groups (*P* < 0.001) (Figure 2).

### 3.5 Analyzing relative differences of marker expression in two research groups

The relative difference of markers expression between the normal and cancerous tissue was measured by ∆∆C*_t_* on Peroxiredoxin‐2, Alpha‐1 antitrypsin, and Haptoglobin.

∆∆C*_t_* was calculated for Peroxiredoxin‐2 mRNA, which indicates the expression of this marker in normal tissue is, on average, 0.94 times cancerous tissue.

∆∆C*_t_* was calculated for Alpha‐1 antitrypsin mRNA, which indicates the expression of this marker in normal tissue is, on average, 0.72 times cancerous tissue.

**FIGURE 3:**
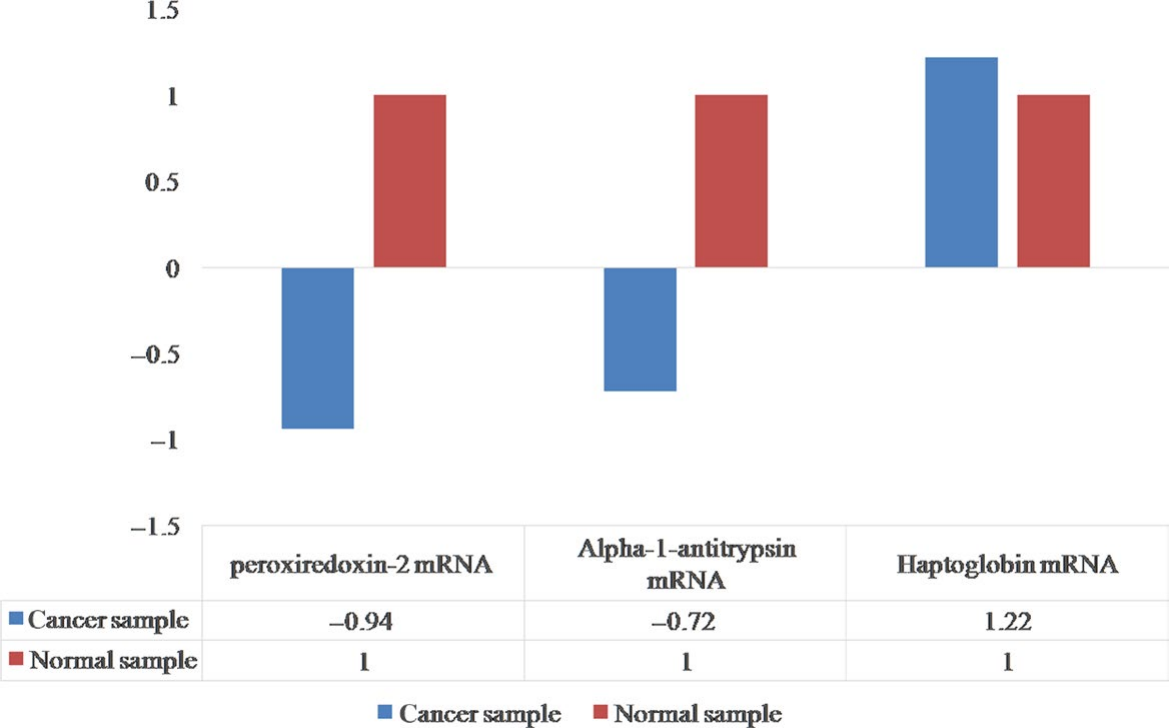
Differences in the expression of Peroxiredoxin‐2 mRNA and Alpha‐1 antitrypsin mRNA and Haptoglobin markers in normal and cancerous tissues

∆∆C*_t_* was calculated for Haptoglobin mRNA, which indicates the expression of this marker in cancerous tissue is, on average, 01.22 times normal tissue. In Figure 3, Differences in the expression of Peroxiredoxin‐2 mRNA and Alpha‐1 antitrypsin mRNA and Haptoglobin markers in normal and cancerous tissues was shown.

## 4 DISCUSSION

The primary of this study was to investigate the quantitative amount of Peroxiredoxin, Haptoglobin, Alpha‐1 Antitrypsin in NSCLC. In the present study the expression of two main proteins of Peroxiredoxin‐2 and Alpha‐1 antitrypsin were downregulated, while the expression of Haptoglobin protein was upregulated in the cancer tissue. LC is one of the leading causes of death in the world and is responsible for an estimated one million deaths per year.^19^ The mortality rate of this cancer is 3 and two times higher than that of prostate and breast cancers in women, respectively. This is worrying because lung cancer is one of the most common malignancies in the world.^20^ LC is the most common cancer among men.^21^ Basal epithelial cell changes and lung epithelium are the origins of more than 90% of LCs.^22^ Only 13% of patients with LC survive 5 years after the diagnosis.^19^ In this study, the identified spots, in accordance with available information in the databases, were Peroxiredoxin‐2 and Haptoglobin, and Alpha‐1 antitrypsin. Peroxiredoxin‐2 protein plays a role in oxidative stress, inflammation, apoptosis regulatory, and inhibition of destructive radicals. This gene encodes a member of the Peroxiredoxin antioxidant enzyme family, which reduces the hydrogen peroxide and alkaloid hydroperoxide. The encoded protein may play an antioxidant protective role in cells and may contribute to the antiviral activity of T8 (+) CD8 cells. This protein may show a proliferative effect and lead to cancer progression. Peroxiredoxin converts organic hydroperoxides into water and alcohol. It plays the role of protecting the cell to deal with oxidative stress by detoxifying peroxides and acts as a sensor of signaling events with hydrogen peroxide. Intracellular H2O2 concentrations may be involved in signaling cascades, growth factors, and tumor necrosis factor.^23^

The amount of this protein was highly reduced in the examined cancer tissue of this study. Forrotta et al (2006) found that PRDX2 is a negative regulator of platelet growth signaling factor, and they have suggested the intervention of quenching it in the melanoma. Among these genes, Peroxiredoxin‐2 (Prdx2) is expressed in natural melanocytes, and its expression in the melanoma is vanished by methylation.^24^

Swaini et al (2006) have found that there is a direct relationship between Peroxiredoxin‐2 expression and tumor malignancy. Low expression of Prdx‐2 in tumor cells makes them more sensitive to oxidative damage. It has been shown that Prdx‐2 protein is not expressed at the early stage of tumorigenesis (in the most of cases) and this activates the malignant tumorigenesis pathways, which indicate this protein may play an important role to prevent the oxidative damage.^25^ It has also shown that Prdx‐2 quenching causes the growth of prostate cancer cells, and blocks the cell cycle at G1 stage. Moreover, the destruction of Prdx‐2 has also decreased the cell growth in Costa Rican resistant prostate cancer cells. Planning purposeful treatment of Prdx‐2 may be a new strategy to develop prostate cancer treatment.^26^ Oxygen‐free radicals, which are generally known as reactive oxygen species (ROS) along with reactive nitrogen species (RNS), are known for their dual role as harmful and useful species.^27^ The "dual" character of ROS within cells acts as secondary messengers in intracellular signal cascades, and lead to cancerous phenotype, however, ROS can also cause cellular aging and apoptosis, and therefore, can act as Antitumorigenic species. The high production of ROS/RNS through internal and external infections is called oxidative stress, and it is suitable to produce many types of cancer cells.^28^ Moreover, the expression of PRDX‐2 in breast cancer cells is linked to a glucose‐dependent phenotype, which is different from bone metastatic cells. In general, their results strongly suggest that PRDX‐2 is a target metabolism, which is protected in the selective growth of metastatic cells in the lungs and protects them to deal with oxidative stress. They suggested that PRXD‐2 protects lung metastatic cells. Peroxiredoxins take part in moving signals to regulate cytochrome release from the mitochondria that is an important step to apoptotic signaling.^29^ Under stress conditions, the PRDX‐2 can migrate from cytosol to the nucleus.

PRDXs, in addition to their antioxidant activities, are involved in various biological functions such as cell proliferation, differentiation, apoptosis, gene expression, and intracellular signaling. PRDX‐2 positively regulates DNA repair.^30^ It seems the loss of PRDX‐2 increases the in vitro cellular aging in adipose fibroblasts.^31^

Haptoglobin is the next identified protein in this study. It is a protein made by the HP in humans. The structure of this protein consists of two alpha and two beta chains, which are linked by disulfide bridges. In the blood plasma, the Haptoglobin‐hemoglobin complex, which is released from erythrocytes, inhibits the oxidative activity of Haptoglobin. The Haptoglobin‐hemoglobin complex is removed by the endoplasmic reticulum (predominantly spleen). This method is used to control the intravascular hemolytic anemia in clinical treatments. In intravascular hemolysis, free hemoglobin is released into the bloodstream, thereby connects Haptoglobin‐hemoglobin, and reduces this Haptoglobin. This protein is often called suicide protein.^32^ Tabassum et al (2012) have found that serum Haptoglobin protein levels increase in many malignant diseases, such as breast cancer. Thus, Haptoglobin probably is a biological prognostic marker for patients with breast cancer.^33^ It has been recently reported that Haptoglobin may be a serologic clinical marker to diagnosis lung adenocarcinoma, especially in men.^10^ Although, the serum level of Haptoglobinis decreased in liver cancer, but it is increased in other cancers.^34^ The obtained data from Haptoglobin show this protein increase in the patients with NSCLC.

We have also found that Alpha‐1 antitrypsin was increased in tumroal tissues compared to normal tissues. This is a glycoprotein and a serine protease inhibitor, which is used to limit the cellular damage and death. It also acts as an acute‐phase protein and is active to control immune and inflammatory proteins, as well as tumor markers.^11^ Yang et al (2008) have concluded that among those who never smoked, SS homozygotes were more at risk for lung cancer. This risk is higher for people exposed to environmental cigarette smoke.^35^ The effect of Alpha‐1 antitrypsin protein on colorectal cancer and its relationship with alpha‐1 antitrypsin deficiency is currently controversial. The only significant statistical findings in their study showed that the serum concentration of this protein is much reduced in the patients with colorectal cancer compared to the control group.^36^ Alpha‐1 antitrypsin deficiency is one of the hereditary features that its main characteristic is the onset of pulmonary emphysema.^37^ Li et al (2011) have reported that Alpha‐1 antitrypsin deficiency significantly increases the risk of lung cancer.^38^

In conclusion, we found that the expression of two major proteins of Peroxiredoxin‐2 and alpha‐1 antitrypsin is dramatically decreased in the cancerous tissues, and the expression level of Haptoglobin in the cancerous tissue is strongly increased. The results of this study showed that there are some differences in term of protein content between the normal and cancerous lung tissues. Further studies are needed to evaluate of these proteins that investigate whether these proteins can candidate as biomarkers to use in the early diagnosis of patients with NSCLC.

### 4.1 Limitations

One of the limitations of this study was providing the control group samples as well as equal and appropriate tumoral tissue from the patients with NSCLC.

## CONFLICT OF INTEREST

It is not declared by the authors.

## AUTHOR'S CONTRIBUTION

AM, RA, and NM designed the study. ZN wrote the manuscript. ZN, MM, AK,HJ, PT, MKPZ, and SS performed experiment and collected the data. SS revised final version of the manuscript. All the authors read and approved the manuscript.

## ETHICS APPROVAL AND CONSENT TO PARTICIPATE

All samples were obtained with patients writing extensively informed consent when they were in hospital and the study was approved by the MasihDaneshvari Hospital ethical committee and conducted in accordance with the ethical guidelines of the Declaration of Helsinki (IR.SBMU.NRITLD.REC.1395.264).

## Data Availability

All data are available by contacting correspondence authors via: mohamadnia.ar@gmail.com.
